# Antioxidants and Health-Beneficial Nutrients in Fruits of Eighteen *Cucurbita* Cultivars: Analysis of Diversity and Dietary Implications

**DOI:** 10.3390/molecules25081792

**Published:** 2020-04-14

**Authors:** Anna Kostecka-Gugała, Michał Kruczek, Iwona Ledwożyw-Smoleń, Paweł Kaszycki

**Affiliations:** Department of Plant Biology and Biotechnology, Faculty of Biotechnology and Horticulture, University of Agriculture in Krakow, al.29 Listopada 54, 31-425 Kraków, Poland; kruczek.michael@gmail.com (M.K.); iwona.ledwozyw-smolen@urk.edu.pl (I.L.-S.)

**Keywords:** pumpkin, squash, Cucurbitaceae, polyphenols, carotenoids, micronutrients, bioactive compounds, antiradical capacity, functional food, aging

## Abstract

Aging is accompanied by gradual accumulation of molecular damage within cells in response to oxidative stress resulting from adverse environmental factors, inappropriate lifestyle, and numerous diseases. Adequate antioxidant intake is a key factor of proper diet. The study aimed to assess the antioxidant/antiradical capacities of *Cucurbita* fruits (18 cultivars of the species: *C. maxima* Duch., *C. moschata* Duch., *C. pepo* L., and *C. ficifolia* Bouché) grown in central Europe. The analyses were based on the FRAP (ferric reducing antioxidant power), CUPRAC (cupric ion reducing antioxidant capacity), and DPPH (2,2-diphenyl-1-picrylhydrazyl radical) assays. The content of phenolic compounds and β-carotene was evaluated with HPLC (high performance liquid chromatography), while the main macro- and micronutrients by ICP-OES (inductively coupled plasma mass spectrometry). The results revealed high intraspecies variability within the *Cucurbita* genus. The Japanese ‘Kogigu’ fruits were distinguished as extraordinary sources of phenolic compounds, including syringic and protocatechuic acids, catechin, and kaempferol. Another popular cultivar ‘Hokkaido’ exhibited the highest antioxidant and antiradical capacities. Most of the fruits proved to be rich sources of zinc and copper. The obtained data are discussed in the context of optimized nutrition of the elderly and suggest that *Cucurbita* fruits should become daily components of their diet.

## 1. Introduction

Pumpkin and squash are common dish ingredients in South America, China, southern Asia, and Japan. They are also used in North American cuisines and are well known in Western Europe. However, in the central and eastern European countries they became partly forgotten and mainly associated with puree juice and soup for the youngest children.

These vegetables belong to the *Cucurbita* genus and they are among the oldest domesticated plants, used as early as ca. 10,000 B.P. [1] on the territory of contemporary Mexico and Guatemala, predating corn and beans by more than 4000 years [1,2]. In the 16th century they were introduced in Europe [3,4] and since then rapidly spread worldwide [5]. Cucurbitaceae family consists of more than 900 species and *Cucurbita* comprises 14 species with six subspecies and two wild varieties [6,7]. The family contains also cucumbers, gourds, melons, watermelons, chilacayotes, and others. The cultivated cucurbits are quite similar in terms of their requirements for growth and development but their fruit morphology (sizes, shapes, colors, pulp structure) is highly variable [2]. Cucurbits are known to reveal wide range of medicinal properties and therefore are recognized as a functional food [8,9,10]. A number of their biologically active compounds were investigated for cytotoxic, hepatoprotective, anti-inflammatory, and cardiovascular properties [11]. For that reason, they may also be regarded as healthy food for healthy aging. It has to be realized that the aging society requires substantial changes in both the nutrient demands and ways of alimentation. It also brings numerous chronically ill patients and leads to increased incidence of comorbidities including hypertension, atherosclerosis, cardiovascular disease, age-related eye diseases, obesity, type II diabetes, neurodegenerative disorders, and cancer. 

The group of the elderly (65+ years old) is growing rapidly and for people aged 80+ their population is predicted to exceed 400 million by 2050 [12]. In 1999, the first “Modified Food Guide Pyramid” for adults aged 70+ was proposed [13]. It was constructed assuming that the narrower base reflects a decrease in energy needs, while emphasizing the importance of nutrient-dense foods, dietary fiber, and water. In addition, nutrient-specific supplements appropriate for older people, e.g., deeply colored fruits and vegetables were recommended. It was highlighted that they should be consumed as a whole fiber-rich food [13]. Recent studies based on HEI-2015 (Healthy Eating Index) [14,15] revealed a positive trend in the nutrition of older Americans. On the contrary, research on the diet of older inhabitants of Switzerland showed that only 38% of this group complied with “The Swiss Food Pyramid” indicating a need for lifelong education in this area [16]. Building social awareness of specific dietary needs of the elderly has become an urgent and challenging task for researchers and physicians. 

All parts of Cucurbitaceae plants are edible and therefore they are grown for seeds, flowers, roots, leaves, and fruits. Flowers (of squash and pumpkins) and roots (of chayote) are ingredients in traditional cuisines [17]. Seeds can be consumed raw or roasted, and may also serve as material for cooking oil, rich in biologically active compounds [18]. In several world regions, *C. pepo* seeds are used in traditional medicine to cure urinary and prostate diseases or as anti-inflammatory, antipyretic, and analgesic remedies. Their antioxidant and lipoxygenase inhibitory activities are well documented [19]. Fruits, in turn, are used when collected at various stages of maturity and can be cooked, baked, pickled, candied or consumed raw. Infusions and decoctions made of *Cucurbita* fruits in traditional medicine are believed to alleviate cold and ache [11,17,20,21]. Note that cucurbits are easily digestible and have soft and delicate textures, which are the features especially important for seniors, particularly the ones with masticatory/swallowing dysfunctions and/or special nutrition needs [12]. The treatment with *C. pepo* fruit pulp extract showed an increase in alkaline phosphatase activity and mucosal thickness which confirmed its gastroduodenal protective and anti-ulcerogenic properties [22]. Pumpkin is considered as a good source of anti-inflammatory substances found helpful in many diseases such as arthritis [23,24].

*Cucurbita* fruits are rich in phenolic compounds: protocatechuic, chlorogenic, salicylic, *p*-hydroxybenoic, *p*-cumaric acids, eriodictyol-7-neohesperidoside, and hesperidin [25]. Foods rich in polyphenols, in particular flavonoids, were shown to modify endothelial formation of NO and to improve endothelium function in humans [26]. Polyphenols were also found to positively influence neuronal cells by attenuating oxidative stress and damage in Alzheimer’s and Parkinson’s diseases as well as in amyotrophic lateral sclerosis [27]. Clinical manifestations of many neurodegenerative diseases are associated with ageing; however, the onset of neuronal death progresses through life [28]. 

Most of anti-cancer properties of *Cucurbita* concern seeds and seed oil; however, several studies revealed anti-carcinogenic potential of fruit-borne compounds. *Cucurbita* polysaccharide named PPPF directly induced apoptosis of HepG2 cells due to down-regulation of the signal transduction pathways, and this mechanism was proposed to facilitate the development of a therapeutic strategy for treating human hepatoma [29]. Cucurbitacins are triterpene secondary metabolites shown to induce apoptosis of various cancer cell lines [17,30] and to arrest the cell cycle at the G2/M phase [31,32]. Note, however, that the role of numerous antioxidants including polyphenols found in *Cucurbita* fruits, in anti-tumor action is still unclear. 

Yellow to dark-orange colors of *Cucurbita* fruits result from high content of carotenoids, mainly β-carotene and/or lutein as well as zeaxanthin [33,34,35]. These carotenoids play an essential role in maintaining ocular health status [36]; β-carotene is a precursor of 11-*cis* retinal, a chromophore of rhodopsin found in rods, receptors enabling vision under low-light conditions. Lutein and zeaxanthin are the main antioxidants of retina, absorbing UV radiation and blue light as well as scavenging free radicals and reactive oxygen species (ROS). The oxidative stress caused by the mentioned radiation contributes to the aging processes resulting, among others, in eye diseases such as age-related macular degeneration (AMD) and cataract [36]. Prevention and treatment of age-related eye diseases includes carotenoid supplementation. 

The common feature of the *Cucurbita* pulp is its low content of fat (about 2.3% in *C. pepo*) [21] and low glycemic index due to the high content of dietary fiber, especially pectins [37]. Cucurbits were shown to reduce the need for insulin in diabetic patients [38]. Many studies confirmed the hypoglycemic efficacy of various polysaccharides found in the pulp [39,40]. Furthermore, the research on animal and human models revealed that treatment with some pumpkin extracts, e.g. *C. moschata*, had hypoglycemic and other anti-diabetic effects as well as stimulated regeneration of pancreatic β-cells [41,42]. *C. ficifolia* (fig-leaf gourd) was even listed within a group of the best anti-obesity medicinal plants due to ability to reduce systemic chronic inflammation accompanying obesity [43]. The results obtained upon the consumption of cucurbit fruits were comparable with those of commonly prescribed anti-diabetic drugs [41]. 

This study was aimed to assess the antioxidant potential and other health-beneficial properties of fruits of 18 cultivars of four species: *Cucurbita maxima* Duchesne, *C. pepo* L., *C. moschata* Duchesne, and *C. ficifolia* Bouché successfully planted under temperate climate conditions of central Europe. The research is expected to expand current knowledge on the health-promoting potential of *Cucurbita* fruits, especially in the context of dietary requirements of the elderly as well as patients suffering from chronic diseases. 

## 2. Results

The highest content of total phenolic compounds (TPC, expressed as chlorogenic equivalents, CAE, in mg per 100 g of fresh weight) was found in the fruits of *C.moschata* ‘Kogigu’ (70.8 mg) (Table 1). That cultivar was also characterized by the highest contents of the following phenolics (given in mg per 100 g of fresh weight): protocatechuic (2.42 mg), syringic (16.41 mg) and ferulic (0.442 mg) acids, catechin (0.52 mg), and kaempferol (0.107 mg). High level of protocatechuic acid was also found in the fruits of ‘Shishigatani’ (1.70 mg) (Table 2 and Figure S1).

Among the analyzed *C. maxima* cultivars, ‘Indomatrone’ and ‘Bambino’ were characterized by a substantially high level of total phenols—i.e., 50.4 and 41.6 mg—respectively. In addition, ‘Indomatrone’ fruits contained one of the highest levels of salicylic acid (2.56 mg), comparable only to the value noted for ‘Table Gold’ of *C. pepo* (2.74 mg). On the other hand, three of the analyzed cultivars, i.e., ‘Chicago Warted Hubbard’, ‘Garbo’, and ‘Triamble’ contained significant amounts of catechin.

For *C. pepo*, the highest levels of total phenolics were noted in the fruits of ‘KamoKamo’ (51.5 mg) and ‘Sweet Dumpling’ (48.1 mg) cultivars. The latter one was also characterized by high accumulation of syringic acid (7.70 mg). The content of total as well as individual phenolic compounds in the fruits of *C. ficifolia* ‘Angel Hair’ was at a relatively low level. 

The highest concentrations of β-carotene (between 12.5–14.6 mg 100 g^−1^f.w.) (Table 1) were found in the fruits of ‘Indomatrone’, ‘Australian Butter’, ‘Chicago Warted Hubbard’ (*C. maxima*), ‘Sweet Dumpling’, and ‘Table Gold’ (*C. pepo*) as well as ‘Kogigu’ and ‘Shishigatani’ (*C. moschata*). Fruits of ‘Musquée de Provence’ (*C. moschata*) and ‘KamoKamo’ (*C. pepo*) contained the lowest levels of that carotene among all analyzed cultivars—i.e., 0.5 and 1.9 mg 100 g^−1^f.w.—respectively. 

The analysis of the antioxidant potential of *Cucurbita* fruits revealed significant differentiation with respect to both the tested cultivar and applied assay (Table 1). The highest values of antioxidant capacity measured by all three assays and expressed as Trolox equivalents, TE, per 100 g of fresh weight were obtained for ‘Hokkaido’ (*C. maxima*) and ‘Angel Hair’ (*C. ficifolia*). Interestingly, pumpkin fruits of ‘Indomatrone’ (*C. maxima*) were characterized by high values of FRAP (85.2 TE) and CUPRAC (256.6 TE) but revealed an exceptionally low antioxidant capacity as measured by the DPPH assay (1.01 TE). Conversely, ‘Musquée de Provence’ (*C. moschata*) fruits exhibited relatively high DPPH values (16.08 TE) and low antioxidant potential as measured by FRAP (31.9 TE) and CUPRAC (86.1 TE) assays when compared to other cultivars. Fruits of ‘Sweet Dumpling’ (*C. pepo*) produced substantially high antiradical scavenging activity (32.10 TE). The lowest values of antioxidant capacity as measured by all three methods were documented for: ‘Butternut’ (*C. moschata*), ‘Halloween’ (*C. pepo*), and ‘Garbo’ (*C. maxima*).

Correlation matrix was constructed to analyze the relation between the content of selected compounds exhibiting antioxidant properties and the values of antioxidant capacities for all the analyzed cultivars (Table 3). A positive correlation between the content of total phenolic compounds and antioxidant capacity as measured by FRAP (*r* = 0.46 *) and CUPRAC (*r* = 0.54 ***) methods was revealed. Contrarily, the content of catechin was negatively correlated with FRAP (*r* = −0.54 ***) and CUPRAC (*r* = −0.44 *) values of pumpkin fruits. Interestingly, the negative relation was also found for concentration of β-carotene (as well as salicylic acid) and antiradical scavenging activity (*r* = −0.65 *** and *r* = 0.53 ***, respectively). The content of syringic acid was positively correlated with that of protocatechuic acid (*r* = 0.64 ***) and total phenols (*r* = 0.78 ***). High value of correlation coefficient for the antioxidant capacities measured by FRAP and CUPRAC (*r* = 0.89 ***) confirms the similarity of the tested methods with respect to the mechanism of action.

The analysis of the content of selected macro- and micronutrients (given per 100 g of fresh weight) revealed substantial diversity between the tested cultivars (Table 4). The ‘Indomatrone’ cultivar (*C. maxima*) revealed the highest accumulation (per 100 gf.w.) of most mineral nutrients, namely: K (469.8 mg), Mg (34.0 mg), S (49.3 mg), Na (6.82 mg), Fe (0.47 mg), and Mn (103.5 mg). At the same time, this cultivar contained relatively low level of B (0.15 mg). Fruits of ‘Sweet Dumpling’ and ‘Table Gold’ of *C. pepo* contained significant amounts of Mg, P, Na, and Fe, while that of ‘Halloween’ (the same species) were particularly rich in calcium (38.0 mg). ‘Kogigu’ (*C. moschata*) fruits were characterized by the highest accumulation of Cu (148.4 µg) as well as of K, P and S. The cultivars containing the lowest levels of mineral elements were ‘Angle Hair’ (*C. ficifolia*), ‘Bambino’ (*C. maxima*), and ‘Miranda’ (*C. pepo*). Interestingly however, in the fruits of ‘Bambino’ a relatively high content of Ca was measured (32.3 mg).

The highest concentration of total soluble sugars (expressed per 100 g of fresh weight) (Table 1) was determined in the fruits of ‘Bambino’ (7.89 g), ‘Indomatrone’ (7.34 g), and ‘Chicago Warted Hubbard’ (7.34 g) cultivars belonging to the *C. maxima* species. For the case of *C. moschata*, only ‘Kogigu’ tended to accumulate high amounts of sugars (6.71 g). The lowest levels were found for ‘Sweet Dumpling’ and ‘Halloween’ (*C. pepo*).

The content of amino acids also showed substantial differences between the analyzed Cucurbitaceae species and cultivars (Table 1). The highest values (in mg per 100 gf.w.) were noted for *C. maxima* cultivars ‘Hokkaido’ (109.5 mg) and ‘Indomatrone’ (71.3 mg), while the lowest for ‘Musquée de Provence’ (7.6 mg, *C. moschata*), ‘Angel Hair’ (13.0 mg, *C. ficifolia*) and ‘Sweet Dumpling’ (12.7 mg, *C. pepo*).

**Figure 1:**
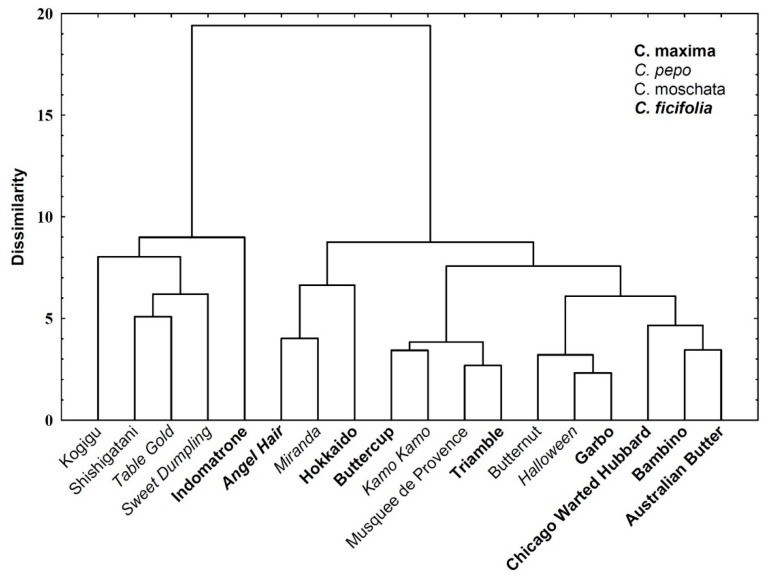
Dendrogram grouping 18*Cucurbita* cultivars based on quality parameters including: the content of total and selected phenolic compounds, antioxidant capacity, the content of mineral elements, amino acids, and soluble sugars in the pulp. Dissimilarity was measured as Euclidean distance and the agglomeration was made with the use of Ward’s method.

The analyzed cultivars were grouped according to their antioxidant and nutritional qualities upon hierarchical cluster analysis (HCA) (Figure 1). The main goal of this attempt was to classify the objects into clusters according to their similarity. Note however, that the employed method did not make it possible to obtain clusters homogenous for individual species of the *Cucurbita* genus.

**Figure 2:**
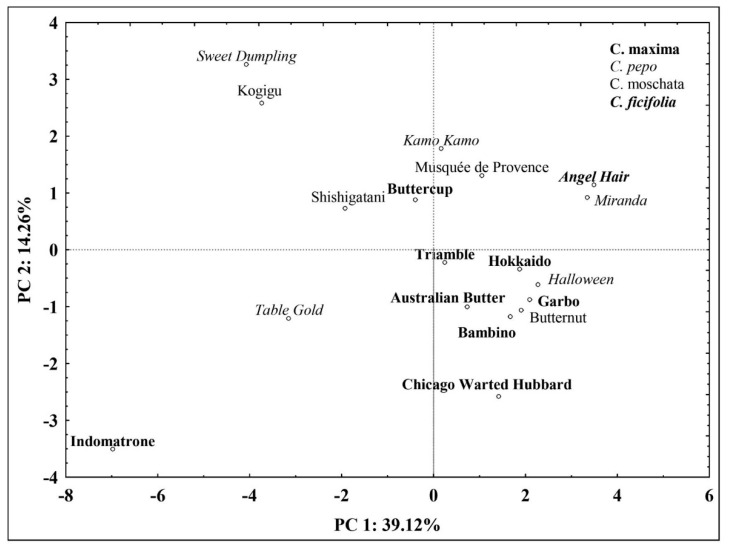
PCA score plot for *Cucurbita* cultivars, PC 1—principal component 1, PC 2—principal component 2.

In the further step, the principal component analysis (PCA) was performed to identify the main sources of variability between the analyzed cultivars with respect to their nutritional qualities (Figure 2). The PCA analysis was applied to mean values and allowed extraction of five principal components with eigenvalues above 1 that accounted for 84.39% of the variability of 21 tested parameters (Table 5). The first principal component (PC1) accounted for 39.12% of the total variance and integrated the content of most mineral elements (Cu, Fe, K, Mg, Mn, Na, P, S, Zn) as well as the total phenolics (Table 6). PC2 (14.26% of total variance) was mainly negatively correlated with the content of salicylic acid. The third principal component (PC3) explained 13.36% of the variance and was positively correlated with the content of amino acids as well as the antioxidant activity as measured by the FRAP method. In turn, PC4 was moderately negatively correlated with the antioxidant capacity measured with all three methods and positively correlated with the content of syringic acid, yet the loading factor did not exceed |0.7|. Finally, PC5 was negatively related to the content of B.

A scatter plot of the score values attributed to the genotypes projected to PC1 and PC2 failed to provide a clear distinction between the analyzed *Cucurbita* species based on the analyzed nutritional and antioxidant parameters. It was mainly because relatively small percentage of variance (53.38%) was explained by the first two principal components (Figure 2). However, most of the tested *C. maxima* cultivars grouped closely with the exception of ‘Indomatrone’. The significant distance of that cultivar from the origin and from other tested genotypes is mainly related to significant accumulation of the analyzed mineral elements, as well as of salicylic and syringic acids as described above. These results indicate high intraspecies variability in *Cucurbita* genus regarding the accumulation of health-promoting compounds and the antioxidant capacity. 

## 3. Discussion

Polyphenols are plant secondary metabolites with high antioxidant capacity. Their activity is determined by direct reaction with free radicals, scavenging of free radicals and singlet oxygen, reactivity as hydrogen- or electron-donating agents, capability of reacting with other antioxidants, ability to produce new generation of antioxidant-derived radicals, and by the potential of chelating transition metals [44,45].Phenolic compounds occur widely in fruits, vegetables, herbs, and beverages [46,47,48,49]. From among the tested *Cucurbita* pulps, the Japanese ‘Kogigu’ (*C. mochata*) cultivar was extraordinary in terms of the total phenolic content; it contained the highest amount of these compounds (70.8 CAE 100 g^−1^f.w.). For syringic acid, the ‘Kogigu’ fruit accumulated this compound three times as much as the second in order ‘Shishigatani’ of the same species. In addition, ‘Kogigu’ contained the most protocatechuic acid and the flavonoids: catechin and kaempferol. The other cultivar with the elevated content of several phenolic acids was *C. maxima* cv. ‘Chicago Warted Hubbard’; its fruits revealed the highest concentrations of *p*-hydroxybenzoic, and *p*-coumaric acids among all the tested fruits, and were the second to accumulate ferulic and caffeic acids.

Biological activity of *p*-coumaric, caffeic, and ferulic acids are quite similar. They are synthesized via the shikimate pathway where *p*-coumaric acid is converted into caffeic acid by hydroxylation, whereas the latter one forms ferulic acid upon methylation [50]. The antioxidant potential of these compounds depends primarily on the number of hydroxyl and methoxy groups attached to the phenyl ring [51]. Antioxidant capacities of a series of phenolic acids and flavonoids were earlier measured by Rice-Evans et al. [44]. Based on their work, the antioxidant power of phenolic acids identified for *Cucurbita* fruits in our study can be arranged in the following order: *p*-coumaric > ferulic > syringic > caffeic > protocatechuic > salicylic >*p*-hydroxybenzoic acids. In turn, according to Kikuzaki et al. [52], the radical DPPH scavenging activity decreased in the order: caffeic acid > ferulic acid >*p*-coumaric acid. In our study, the contents of *p*-coumaric and caffeic acids in all samples were relatively low, while the contents of ferulic acid varied significantly upon the tested cultivar. The fruits of ‘Kogigu’ (*C. moschata*), ‘Garbo’, and ‘Chicago Warted Hubbard’ (*C. maxima*) contained the highest amounts of ferulic acid. Cucurbits are known as rich sources of this polyphenol compared to other fruits and vegetables; only some leguminous vegetables and tomatoes were shown to accumulate it to a greater extent [53]. Ferulic acid is known to exert anti-angiogenic and anti-tumor effects by affecting the activity of vascular endothelial growth factor (VEGF), platelet derived growth factor (PDGF) and hypoxia-inducible factor 1 (HIF-1) [54]. It can also inhibit skin photo-aging and therefore has been used as a component of cosmetic preparations [53]. Salicylic acid was also detected in the examined *Cucurbita* fruits. Notwithstanding its moderate antioxidant capacity, this compound is widely known to reduce the risk of myocardial infarction and ischemic stroke. Although its content in cucurbits is much lower than e.g., in raspberries, the pumpkin pulp can still provide continuous supplementation in this phenolic compound due to the long storage time and the substantially larger resources available for food processing.

Two flavonoids: catechin and kaempferol were detected in most fruit pulps. Flavonoids are efficient antioxidants with well-pronounced health-beneficial properties. They can protect against oxidative-stress based diseases and are able to modulate enzyme activities as well as interactions with specific receptors. Their comprehensive mode of action includes quenching of free radicals, chelating metals, suppressing the enzymes associated with free radical generation, and stimulation of internal antioxidant enzymes [55]. Protective action of flavonoids against cardiovascular diseases was confirmed, which was based—among other mechanisms—on decreasing the oxidation rate of low-density lipoproteins [56]. Kaempferol has recently focused special interest and is currently considered as a potential cancer treatment agent because of its strong capacity to reduce the oxidative stress [57,58]. Note that, although in the commonly consumed vegetables (e.g., onions) and in several herbs, kaempferol may occur in concentrations much higher than in pumpkin, the size of a single serving of a pumpkin dish is significantly larger and therefore may provide considerable and biologically-significant quantities of this health-beneficial compound. 

The obtained results indicate that *Cucurbita* vegetables contain relatively low amounts of phenolics, especially when compared to richer sources such as berries or grapes [46,59]. However, the level of phenolic compounds in selected *Cucurbita* cultivars was comparable to those noted for potatoes (36.9–52.7 mg 100 g^−1^f.w.) [60] and carrots, with the exception of purple cultivars (24.2–40.4 mg 100 g^−1^f.w.) [61]. Additionally, the pulp of selected *Cucurbita* cultivars, such as ‘Indomatrone’ or ‘Sweet Dumpling’ can be considered as a richer source of essential mineral nutrients such as: Cu, Zn, and Mg as compared to the mentioned two other crop species [62,63].

It should be emphasized that the abundance of phenolic compounds does not necessarily imply high total antioxidant potential of any biological sample. The antioxidant properties of *Cucurbita* may also result from the presence of carotenoids which are usually assumed to play a predominant role in this respect. However, the pumpkin pulp was also found to be rich in vitamins C and E [64,65] as well as in carbohydrates, which all might add to the resultant activity. The abovementioned facts imply that it is very difficult to estimate the total antioxidant power only based on the content of particular bioactive compounds. This conclusion gave reasons to launch direct measurements of antioxidant (CUPRAC, FRAP) and antiradical (DPPH) capacities of pumpkin flesh extracts. For the case of Cucurbitaceae, it is a novel approach, first applied in our pilot work [66], and follows that of the most recent study of Kulczyński et al. [67]. Similarly to our strategy, these authors have employed several independent methods to evaluate antioxidant potential of 14 *C. maxima* cultivars. Their optimized extraction method (with 80% methanol/water) was very close to the one elaborated for this study. Here, apart from *C. maxima* (out of the eight cultivars, only ‘Hokkaido’ and ‘Buttercup’ were examined in both studies) the testing included also cultivars belonging to other species (*C. pepo*, *C. moschata*, *C. ficifolia*). 

The fruit of ‘Hokkaido’ exhibited the maximum antioxidant potential as revealed by the assays CUPRAC, FRAP, and DPPH which are the most sensitive methods towards phenolic compounds [59]. Positive correlations between TPCs and the antioxidant capacities were also shown in this work (Table 3). This cultivar is famous for its intensive orange-colored pulp, which results from the high concentration of carotenoids, mainly β-carotene [33,34] whose antioxidant activity is based on singlet oxygen quenching and ability to trap peroxyl radicals [68]. Taking into account the content of phenolic compounds (Table 2), it is thus justified that both carotenoids and phenolics contributed to the resultant exceptional activity. It should be noted here that in the complementary study [67] ‘Hokkaido’ also ranked high among the other tested *C. maxima* cultivars in terms of antioxidative/antiradical activity. The fruits of ‘Indomatrone’ accumulated the highest amounts of total phenols and, expectedly, they revealed the highest antioxidant capacities as measured by FRAP and CUPRAC assays. At the same time, their antiradical capacity determined upon the DPPH method was the lowest of all the tested fruits. Possibly, the pulp of ‘Indomatrone’ contained some fraction of other polyphenols with a different mechanism of action than the typical polyphenols of *Cucurbita*. Note that, in fact, only the DPPH assay allowed for direct measurement of antiradical capacity since DPPH itself is a free radical. Also, high content of soluble sugars as measured for this cultivar should be considered as a reliable explanation of the observed facts. It was demonstrated that the mono- and disaccharides, especially fructose, interfered with the Folin-Ciocalteu reagent, leading to overestimation of the final results [69]. Hence, the antioxidants of ‘Indomatrone’ fruits require a more detailed analysis.

Many fruits and leaves rich in lutein are considered helpful in treatment of aged-related macular degeneration (AMD) and cataracts as the loss of these pigments in the retina is observed during the AMD development. However, supplementation with sole lutein or with lutein and zeaxanthin had little or no effect on progression of AMD and subsequent AMD vision loss, while additional zinc combined with the antioxidant vitamins (C and E) slowed down progression of this disease [70]. *C. pepo* ‘Sweet Dumpling’ and *C. maxima* ‘Indomatrone’ were the cultivars with the highestZn content in their fruits (1.23 and 0.88 mg 100 g^−1^ f.w., respectively). Note that these levels can cover from 8 to 15% of zinc Recommended Daily Allowance (RDA) for people over 70 years old (Table S1) [71,72]. The two cultivars were also distinguished in the high content of β-carotene (14.6 and 14.8 mg 100 g^−1^f.w., respectively) which is a precursor of zeaxanthin. Since the *Cucurbita* fruits contain pronounced amounts of vitamin E [21,64,65] as well as these xanthophylls [64,65], they seem to have the necessary qualities to slow down the development of the retinal diseases.

Trace elements in human nutrition are required for the proper activity of antioxidant enzymes crucial for efficient defense against the excess of reactive oxygen species (ROS): Cu, Mn, and Zn for superoxide dismutase (SOD), Fe for catalase (CAT), and Se in selenocysteine for glutathione peroxidase (GPx). Our data show that the tested *Cucurbita* fruits contained significant amounts of Zn and Cu as referred to RDA values for seniors (Table S1) [71,72]. The highest Cu content was noticed for ‘Kogigu’ of *C. moschata*; its 100 g serving allows for 16.5% supply of daily demand for this microelement. It was confirmed that Zn supplementation effectively reduced oxidative stress and generation of inflammatory cytokines such as TNF-α and IL-1β in elderly individuals [73]. Furthermore, the relationship between Cu to Zn ratio (CZr) and mortality rates gave reasons to suggest that CZr is a biomarker of aging.

The presented research work on fruits of 18 *Cucurbita* cultivars enabled to compare their antioxidant properties and confirmed the great diversity found for different objects. This observation is similar to that reported by other authors for many other examined cultivars [64,65,67]. Such variability should not be considered surprising, taking into account that pumpkin is one of the first domesticated plants and has been grown worldwide for several hundred years thus giving farmers enough time to obtain and introduce cultivars with unique characteristics. The fruits subjected to our study differed profoundly in terms of the content of phytochemicals as well as their antioxidant potential and antiradical capacity. Statistical analyses show that these differences were associated with distinct characteristics of a particular cultivar, and could not be generalized as specific to individual species. The greatest variations in phenolic content were reported for protocatechuic, syringic, and salicylic acids. Moreover, not all the tested phenols were detected in all the cultivars. Unfortunately, the majority of other available studies on *Cucurbita* do not bring results as dependent on examination of several variant cultivars, which leaves little space for making comparisons. 

Generally, it is difficult to compare the data on the content of antioxidants in the fruits of different cultivars. The resultant information is influenced by genetic differences and affected by environmental conditions, degree of maturity at harvest, and storage conditions [74]. Note that *Cucurbita* fruits can often be used when they are not yet fully ripe. *C. ficifolia*, *C. maxima*, and *C. moschata* are known as winter squashes that can be stored for months; however, among the *C. pepo* cultivars there are several ones whose fruits can be consumed only as summer squashes. Fruit maturation involves a series of complex reactions that lead to changes in plant phytochemistry. Two different phenomena of phenolic compound changes were observed during maturation: a gradual decrease [75,76] or an increase at the end of the process [77,78,79]. Furthermore, concentration of antioxidants varied within the plant organs and tissues [80,81].

In many research articles, it is stressed that high reactivity and great structural and molecular diversity of phenolics makes them very difficult to study, as evidenced by ambiguous and sometimes contradictory results [82]. Divergent sample preparation procedures and the lack of measurement standardization are additional reasons for unsatisfactory data comparability. Therefore, we emphasize the need for methodological unification of antioxidant capacity tests based on standardized extraction procedures, especially regarding groups of related plants with similar chemical characteristics. We also point to the fact that extensive studies on beneficial action of phenolic compounds on human health have only started a few years ago [27]. It is already known that dietary polyphenols undergo several transformations in the body (i.e., deglycosylation, oxidation, dehydroxylation, demethylation), and their bioavailability may vary significantly [82], usually remaining relatively low [83]. Therefore, the elevated and potentially toxic concentrations of polyphenols, as reported in several works, can only be reached when these compounds are applied as concentrated supplements or therapeutic medicines [28]. Unfortunately, the data on the bioavailability of phenolics and other nutrients of *Cucurbita* fruits are still scarce. Studies on the content of anti-nutrients such as tannins, oxalates, saponins, phytates, alkaloids, and cyanide in *Cucurbita* pulp, demonstrate low or acceptable amounts of these substances as referred to the daily intake [35,84,85,86]. Considering the above, the application of polyphenols obtained from the natural sources such as cucurbits appear to be the most favorable and the safest way of supplementation. 

As regards directions of future studies of natural antioxidative agents, the research should focus on characterizing antioxidant intake mechanisms and correlating them with biological availability. This approach seems to be necessary to complement current efforts made to elucidate the impact of antioxidants on oxidative stress responses. Also it is noteworthy that the antioxidant capacity analyses of this study were performed in vitro and, although the standardized methods were employed, the next-stage complementary research is required involving the in vivo testing with free radicals physiologically present in the human body. Moreover, appropriate clinical trials are necessary to evaluate real dietary potential of pumpkin fruits towards the elderly population. 

To conclude, the results obtained from a systematic research on fruits of 18 *Cucurbita* cultivars bring valuable information on the unique properties of *Cucurbita* and reveal considerable diversity of both the content of bioactive compounds and antioxidant/antiradical capacities. The detected phenolic compounds, β-carotene, Zn, and Cu could be useful in the nutrition of elderly people suffering from chronic diseases. It is finally emphasized, however, that even if the concentration of a particular nutrient or antioxidant is lower than in other plant material (medicinal plants, herbs, other vegetables), the *Cucurbita* fruits have the advantage of being easily digestible and possessing low glycemic index, which allows for their supply in high amounts and serving in many variant ways after appropriate processing. 

## 4. Materials and Methods

### 4.1. Plant Material

All studied fruits were harvested in Wawrzeńczyce (50°06′N20°19′E; southern Poland). Eight cultivars were of *Cucurbita maxima* Duchesne species (‘Australian butter’, ‘Bambino’,‘Buttercup’,‘Chicago Warted Hubbard’, ‘Garbo’, ‘Hokkaido’, ‘Indomatrone’, and ‘Triamble’), five cultivars of *Cucurbita pepo* L. (‘Halloween’, ‘KamoKamo’, ‘Miranda’, ‘Sweet Dumpling’, and ‘Table Gold’), four cultivars of *Cucurbita moschata* Duchesne (‘Butternut’, ‘Kogigu’, ‘Musquée de Provence’, and ‘Shishigatani’) and one (‘Angel Hair’) was a cultivar of a fig-leaf gourd (*Cucurbita ficifolia* Bouché). The fruits were fully ripe and were examined immediately after harvesting. Upon collecting, the fruit material was fragmented into small pieces, frozen at −25 °C and then freeze-dried (0.37 mBa) for 48 h. The samples were stored at −25 °C in the dark. 

### 4.2. Sample Preparation

The extracts were prepared by grinding 0.75 g of the freeze-dried material in a mortar with 80% methanol (Sigma–Aldrich, St. Louis, MO, USA) applied in several portions. Each homogenate was then filtered through a sintered glass funnel to a volumetric flask and filled up to the total volume of 25 mL. The extraction procedure was carried out at 22 °C under limited lighting. Three fruit extracts were made for each cultivar. The extracts were stored in the dark at −25 °C for a maximum of two weeks.

### 4.3. Total Phenolic Content (TPC)

The content of phenolic compounds in the extracts was determined based on the reaction with the Folin–Ciocalteu reagent [87]. The extract (0.25 mL) was mixed with 0.25 mL of 25% Na_2_CO_3_, 0.125 mL of the Folin–Ciocalteu reagent (Sigma–Aldrich, diluted twice with water prior to the analysis), 2.25 mL of water, and then incubated for 15 min. The absorbance was measured at 760 nm (JASCO V-530 UV–Vis spectrophotometer). The final results were expressed as mg of chlorogenic acid (Sigma–Aldrich) per 100 g of fresh weight (chlorogenic acid equivalents, CAE 100 g^−1^).

### 4.4. Antioxidant Capacity—A FRAP Assay

The FRAP (ferric reducing antioxidant power) assay is based on the reduction of ferric–tripyridyl-*s*-triazine (Fe^+3^–TPTZ) complex to its ferrous derivative (Fe^2+^) [88]. The FRAP working solution was prepared fresh by mixing 300 mM acetate buffer (pH 3.6), 10 mM TPTZ (Sigma–Aldrich) in 96% ethanol, and 20 mM FeCl_3_ (10:1:1, *v*:*v*:*v*). Then, 3 mL of FRAP working solution were mixed with 0.1 mL of fruit extract and 0.3 mL of water. The absorbance was measured at 595 nm after 30 min. The results were expressed as μmol Trolox (6-hydroxy-2,5,7,8-tetramethylchroman-2-carboxylic acid; Sigma–Aldrich) per 100 g of fresh weight (Trolox equivalents, TE 100 g^−1^).

### 4.5. Antioxidant Capacity—A CUPRAC Assay

The CUPRAC (cupric ion reducing antioxidant capacity) assay is based on the measurement of utilization of copper (II)-neocuproine as chromogenic oxidizing agent [89,90,91]. Briefly, 1 mL of 10 mM CuCl_2_, 1 mL of 7.5 m Mneocuproine (Sigma–Aldrich) in 96% ethanol and 1 mL of 1 M NH_4_Ac buffer, pH 7.0, were mixed with 0.3 mL of the fruit extract and 0.8 mL of water. The absorbance was measured at 450 nm after 30 min. The results were expressed as μmol Trolox (Sigma–Aldrich) per 100 g of fresh weight (TE 100 g^−1^).

### 4.6. Radical Scavenging Capacity (RSC)–A DPPHAssay 

The radical scavenging capacity of extracts was tested following the reduction of a synthetic, stable free radical 2,2-diphenyl-1-picrylhydrazyl (DPPH•). The colorimetric method enables to measure absorbance changes of DPPH solution at 517 nm as a result of antioxidant activity of the sample [92]. Briefly, 2.8 mL of 0.1 mM DPPH (Sigma–Aldrich) solution in 96% ethanol was mixed with 0.2 mL of the extract. The DPPH absorbance was detected after 30 min. The RSC results were expressed as μmol Trolox (Sigma–Aldrich) per 100 g of fresh weight (TE 100 g^−1^).

### 4.7. Identification of Phenolic Compounds with HPLC

In order to identify phenolic compounds in fruit extracts, a high-performance liquid chromatography (HPLC) method was used (Shimadzu LC–10AS chromatograph equipped with a C18 RP column and SPD-10AV UV–Vis detector). Signal detection was set at the wavelengths of 325 and 265 nm. Chromatographic separation was carried out at 33 ± 1 °C. using the following solvents: (A) water (Sigma–Aldrich) with acetic acid (0.1%), (B) methanol (Sigma–Aldrich, ultra pure) with acetic acid (0.1%) and applying the gradient: 90% A, 10% B for 20 min; 75% A, 25% B for 30 min; 65% A, 35% B for 40 min; 55% A, 54% B for 50 min; 50% A, 50% B for 60 min; 30% A, 70% B for 62 min; 100% B to 80 min; 80% A, 10% B up to 85 min. The flow rate was 1 mL/min. The identification of phenolics was based on the retention times of chlorogenic, caffeic, *p*-hydroxy-benzoic acid, *p*-coumaric, ferulic, protocatechuic, syringic acids (Sigma–Aldrich), salicylic acid (LGC Standards) as well as (+)-catechin (LGC Standards) andkaempferol (Sigma–Aldrich).

### 4.8. Identification of β-Carotene with HPLC

Identification of β-carotene was carried out employing the high performance liquid chromatography method (Shimadzu LC–20AD chromatograph with Develosil RPAQEOUS-AR C30 column and Shimadzu SPDM–20A–DAD photodiode-array detector). The signal detection was set at the wavelengths of 452 and 444 nm. The separation was carried out at 25±1 °Cwith the solvents: (A) 1% water in methanol (Sigma–Aldrich, ultra pure), (B) methanol, (C) 10% *n*-hexane (Sigma–Aldrich, ultra pure) in acetonitrile (Sigma–Aldrich, ultra pure), and applying the following gradient: starting with the initial ratio of 95% A, 5% B to 30% A, 70% B up to 5 min; then 100% B for 10 min; 100% C up to 25 min, then maintaining this proportion up to 60 min; 100% B up to 62 min; 95% A, 5% B up to 63 min and maintaining this proportion up to 70 min separation. The flow rate was 1 mL/min. The identification of β-carotene was based on the retention time of a standard compound (Sigma–Aldrich) and confirmed by analysis of absorption spectra. β-carotene was determined for selected fruits only, namely for these cultivars that had not earlier been tested and documented in the literature data.

### 4.9. Determination of the Content of Selected Macro- and Microelements, Free Amino Acids, and Soluble Sugars 

The content of Ca, K, Mg, P, S, B, Cu, Fe, Mn, Na, and Zn in the pulp of *Cucurbita* cultivars was measured with an ICP-OES technique. Samples of 0.5 g of freeze-dried pulp were subjected to microwave digestion in 65% super-pure nitric acid with the use of CEM MARS-5 Xpress (CEM World Headquarters, Matthews) microwave digestion system [93]. Determination of soluble sugars and free amino acids was conducted in methanolic extracts of the freeze-dried pulp. The content of soluble sugars was measured with the anthrone method using glucose as a standard [94]. Free amino acids were analyzed spectrophotometrically after the reaction of methanolic extracts with ninhydrin using glycine as a standard compound [95]. The obtained results were calculated per 100 gf.w.

All other chemicals such as salts and buffer constituents were of analytical grade, purchased from Chempur/POCh (Poland). All standards and solutions were prepared with p.a. chemicals and deionized water daily prior to the measurements. During analyses of antioxidant compounds, the room was partially darkened and a stable room temperature of 22 °C provided. The analytical spectroscopic system was maintained according to the GLP rules [96].

### 4.10. Statistical Analysis

All analyses were performed in triplicates. One-way analysis of variance (ANOVA) was applied to compare differences between mean values and the means were compared by a Duncan’s test at α = 0.05. Calculations of Pearson’s correlation coefficients for selected parameters were performed. To overview the correlations between quality parameters as well as the relationships between Cucurbitaceae cultivars and their chemical characteristics, a PCA analysis was performed on the correlation matrix obtained for the standardized data set [97]. The hierarchical cluster analysis (HCA) was carried out based on the Euclidean distance and Ward’s method. All statistical analyses were done with the use of Statistica 13 software (StatSoft) [98].

## Figures and Tables

**Table 1:** Antioxidant capacity and the content of β-carotene, phenolic compounds, soluble sugars, and free amino acids in the pulp of 18*Cucurbita* cultivars.

		Antioxidant Capacity			Content			
		TE 100 g^−1^ f.w.			mg 100 g^−1^f.w.	CAE 100 g^−1^f.w.	g 100 g^−1^f.w.	mg 100 g^−1^f.w.
Species	Cultivar	CUPRAC	FRAP	DPPH	β-Carotene	Total phenols	Soluble sugars	Amino acids
***C. maxima***	Australian Butter	193.6 ± 11.2 ^g^	50.9 ± 3.5^ef^	2.90 ± 0.50 ^abc^	13.1 ± 0.5 ^cde^	19.7 ± 0.9 ^abc^	6.43 ± 0.38 ^e^	57.8 ± 1.2 ^e^
	Bambino	118.4 ± 10.6 ^de^	40.5 ± 9.8 ^cde^	13.07 ± 2.27 ^def^	n.a.	41.6 ± 4.2 ^fgh^	7.89 ± 0.29 ^g^	63.4 ± 1.7 ^ef^
	Buttercup	130. ± 19.3 ^e^	41.3 ± 1.8 ^cde^	5.76 ± 1.00 ^abc^	n.a.	36.0 ± 2.8 ^efg^	4.30 ± 0.18 ^bcd^	35.3 ± 0.1 ^d^
	Chicago Warted Hubbard	109.3 ± 3.4 ^bcde^	33.6 ± 1.3 ^bcd^	8.86 ± 1.54^cde^	12.7 ± 0.6 ^cde^	18.9 ± 2.0 ^abc^	6.90 ± 0.09^ef^	64.2 ± 5.1 ^ef^
	Garbo	99.4 ± 3.8 ^bcd^	22.3 ± 0.8 ^ab^	1.58 ± 0.27 ^ab^	11.9 ± 0.5 ^cd^	27.6 ± 1.8 ^bcde^	4.36 ± 0.35 ^bcd^	23.1 ± 2.3^bc^
	Hokkaido	251.5 ± 20.3 ^h^	139.9± 13.1 ^i^	32.46 ± 1.06^i^	n.a.	20.6 ± 0.5 ^abc^	6.10 ± 0.62 ^e^	109.5 ± 6.7 ^g^
	Indomatrone	256.6 ± 2.6 ^h^	85.2 ± 2.9 ^h^	1.01 ± 0.03 ^a^	14.8 ± 0.9 ^e^	50.4 ± 3.1 ^h^	7.34 ± 0.25^fg^	71.3 ± 7.0 ^f^
	Triamble	114.7 ± 3.7 ^cde^	21.2 ± 3.6 ^ab^	3.57 ± 0.62 ^abc^	11.2 ± 0.2 ^c^	32.5 ± 1.9 ^def^	4.97 ± 0.03 ^d^	27.2 ± 2.2 ^cd^
***C. pepo***	Halloween	64.3 ± 3.6 ^a^	11.7 ± 1.7 ^a^	3.31 ± 0.57 ^abc^	11.5 ± 0.2 ^c^	21.3 ± 2.2 ^abcd^	3.03 ± 0.27 ^a^	25.9 ± 1.6 ^cd^
	Kamo Kamo	168.8 ± 1.4 ^fg^	57.8 ± 3.8 ^f^	21.03 ± 3.65 ^gh^	1.9 ± 0.0 ^a^	51.5 ± 11.0 ^h^	3.64 ± 0.26 ^abc^	15.5 ± 1.2 ^ab^
	Miranda	102.7 ± 6.8 ^bcde^	33.0 ± 0.8 ^bcd^	13.92 ± 0.45 ^ef^	7.6 ± 0.8 ^b^	13.2 ± 0.6 ^a^	6.35 ± 0.36 ^e^	22.6 ± 0.9 ^bc^
	Sweet Dumpling	178.6 ± 16.1 ^fg^	51.2 ± 3.1 ^ef^	32.10 ± 5.56 ^i^	14.6 ± 0.6 ^e^	48.1 ± 1.8 ^h^	2.78 ± 0.13 ^a^	12.7 ± 2.5 a
	Table Gold	173.1 ± 8.1 ^fg^	33.4 ± 3.1 ^bcd^	5.64 ± 0.98 ^abc^	14.4 ± 0.3 ^e^	29.8 ± 3.1 ^cde^	4.38 ± 0.05 ^bcd^	28.1 ± 1.2 ^bc^
***C. moschata***	Butternut	83.1 ± 4.7 ^ab^	21.7 ± 2.5 ^ab^	2.45 ± 0.35 ^ab^	11.1 ± 0.6 ^c^	17.1 ± 1.1 ^ab^	4.49 ± 0.13 ^cd^	64.7 ± 3.7 ^ef^
	Kogigu	158.8 ± 2.0 ^f^	34.7 ± 2.4 ^bcd^	7.78 ± 1.35 ^bcd^	14.3 ± 2.3 ^de^	70.8 ± 6.3 ^i^	6.71 ± 0.36 ^ef^	59.5 ± 3.2 ^e^
	Musquéede Provence	86.1 ± 1.9 ^abc^	31.9 ± 1.7 ^bc^	16.08 ± 2.79 ^fg^	0.5 ± 0.1 ^a^	29.2 ± 0.6 ^cde^	3.50 ± 0.04 ^ab^	7.6 ± 0.5 ^a^
	Shishigatani	194.8 ± 9.1 ^g^	47.9 ± 4.6 ^def^	15.01 ± 1.02 ^f^	12.5 ± 0.6 ^cde^	46.6 ± 2.1 ^gh^	4.66 ± 0.23 ^d^	56.2 ± 2.7 ^e^
***C. ficifolia***	Angel Hair	233.3 ± 4.7 ^h^	71.4 ± 4.4 ^g^	24.52± 0.86 ^h^	n.a.	20.6 ± 0.1 ^abc^	4.56 ± 0.24 ^d^	13.0 ± 0.2 ^a^

The values are given as means ± standard errors, followed by the letters a–i (in superscripts) to indicate statistical significance. The values marked with the same letters in one column are not statistically different at the *p* < 0.05; *n* = 3; n.a., not analyzed.

**Table 2:** Content of selected phenolic compounds in the pulp of 18*Cucurbita* cultivars.

		Content (mg 100 g^−1^f.w.)									
Species	Cultivar	Protocatechuic Acid	*p*-hydroxy-Benzoic Acid	Catechin	Chlorogenic Acid	Caffeic Acid	*p*-Coumaric Acid	Syringic Acid	Ferulic Acid	Salicylic Acid	Kaempferol
***C. maxima***	Australian Butter	0.69 ± 0.09 ^d^	0.003 ± 0.001	0.06 ± 0.01 ^abc^	n.d.	n.d.	0.02 ± 0.003	1.29 ± 0.03 ^abc^	0.012 ± 0.000	1.39 ± 0.12 ^e^	0.025 ± 0.002
	Bambino	0.09 ± 0.00 ^ab^	0.007 ± 0.000	0.15 ± 0.02 ^bc^	n.d.	0.03 ± 0.00	0.02 ± 0.004	4.43 ± 0.26 ^ef^	n.d.	1.43 ± 0.30 ^e^	n.d.
	Buttercup	0.36 ± 0.02 ^c^	0.001 ± 0.000	0.03 ± 0.01 ^a^	n.d.	0.05 ± 0.00	n.d.	2.04 ± 0.24 ^bc^	n.d.	0.10 ± 0.02 ^a^	0.046 ± 0.001
	Chicago Warted Hubbard	0.29 ± 0.05 ^bc^	0.027 ± 0.000	0.38 ± 0.03 ^g^	n.d.	0.05 ± 0.02	0.03 ± 0.004	1.46 ± 0.12 ^abc^	0.231 ± 0.060	1.76 ± 0.15 ^f^	0.042 ± 0.009
	Garbo	0.52 ± 0.01 ^cd^	0.004 ± 0.001	0.42 ± 0.04 ^g^	n.d.	n.d.	0.03 ± 0.004	4.54 ± 0.21 ^ef^	0.259 ± 0.011	1.66 ± 0.09 ^ef^	0.056 ± 0.006
	Hokkaido	1.16 ± 0.02 ^e^	0.001 ± 0.000	0.02 ± 0.00 ^a^	n.d.	0.04 ± 0.00	n.d.	0.62 ± 0.03 ^ab^	0.020 ± 0.004	0.68 ± 0.02 ^bc^	0.042 ± 0.003
	Indomatrone	0.66 ± 0.01 ^d^	0.020 ± 0.002	0.06 ± 0.00 ^ab^	n.d.	n.d.	0.02 ± 0.003	4.25 ± 0.19 ^ef^	0.020 ± 0.002	2.56 ± 0.12 ^g^	0.060 ± 0.009
	Triamble	0.03 ± 0.00 ^a^	0.006 ± 0.000	0.37 ± 0.01 ^g^	0.03 ± 0.00	n.d.	0.01 ± 0.002	2.77 ± 0.10 ^cd^	0.143 ± 0.014	1.49 ± 0.11 ^ef^	n.d.
***C. pepo***	Halloween	1.07 ± 0.10 ^e^	0.002 ± 0.001	0.22 ± 0.02 ^ef^	0.14 ± 0.02	0.03 ± 0.01	0.01 ± 0.001	2.18 ± 0.05 ^bc^	0.010 ± 0.001	1.06 ± 0.04 ^d^	0.027 ± 0.003
	KamoKamo	1.08 ± 0.08 ^e^	0.002 ± 0.000	0.15 ± 0.02 ^cde^	n.d.	0.01 ± 0.00	n.d.	4.91 ± 0.16 ^ef^	0.025 ± 0.001	0.61 ± 0.03 ^bc^	0.022 ± 0.001
	Miranda	1.46 ± 0.06 ^f^	0.001 ± 0.000	0.09 ± 0.00 ^abc^	0.02 ± 0.00	0.04 ± 0.00	0.02 ± 0.002	1.47 ± 0.14 ^abc^	0.015 ± 0.003	0.43 ± 0.04 ^b^	n.d.
	Sweet Dumpling	0.42 ± 0.03 ^c^	0.002 ± 0.000	n.d	0.06 ± 0.00	n.d.	0.01 ± 0.000	7.70 ± 0.72 ^g^	0.072 ± 0.013	0.12 ± 0.01 ^a^	n.d.
	Table Gold	1.03 ± 0.11 ^e^	0.004 ± 0.000	0.19 ± 0.00 ^de^	n.d.	n.d.	0.01 ± 0.003	2.14 ± 0.08 ^bc^	n.d.	2.74 ± 0.09 ^g^	n.d.
***C. moschata***	Butternut	1.03 ± 0.07 ^e^	0.009 ± 0.000	0.28 ± 0.01 ^f^	n.d.	n.d.	0.02 ± 0.00	0.84 ± 0.05 ^ab^	0.196 ± 0.009	0.82 ± 0.02 ^cd^	0.048 ± 0.008
	Kogigu	2.42 ± 0.20 ^h^	0.014 ± 0.000	0.52 ± 0.06 ^h^	n.d.	0.04 ± 0.00	0.03 ± 0.006	16.41 ± 1.77 ^h^	0.442 ± 0.005	0.50 ± 0.04 ^bc^	0.107 ± 0.043
	Musquéede Provence	0.32 ± 0.06 ^bc^	0.003 ± 0.000	n.d.	n.d.	n.d.	n.d.	3.72 ± 0.51 ^de^	n.d.	0.50 ± 0.13 ^bc^	0.026 ± 0.003
	Shishigatani	1.70 ± 0.15 ^g^	0.015 ± 0.000	0.13 ± 0.03 ^bcde^	0.03 ± 0.00	0.08 ± 0.01	0.02 ± 0.001	5.42 ± 0.36 ^f^	n.d.	0.45 ± 0.02 ^b^	n.d.
***C. ficifolia***	Angel Hair	0.27 ± 0.02 ^bc^	0.004 ± 0.000	0.12 ± 0.00 ^bcd^	n.d.	0.03 ± 0.00	n.d.	0.39 ± 0.06 ^a^	n.d.	0.04 ± 0.00 ^a^	n.d.

The values are given as means ± standard errors, followed by the letters a–h (in superscripts) to indicate statistical significance. The values marked with the same letters in one column are not statistically different at the *p* < 0.05; *n* = 3; n.a., not analyzed. The order of the compounds corresponds to the order of retention times (HPLC).

**Table 3:** Correlation matrix for selected antioxidant parameters of fruit pulp of *Cucurbita* cultivars.

	TP	FRAP	CUPRAC	DPPH	Car	PcA	pHbA	SyrA	SA	Cat
TP	1.00									
FRAP	0.46 *	1.00								
CUPRAC	0.54 ***	0.89 ***	1.00							
DPPH	0.32	0.22	0.10	1.00						
Car	0.05	−0.03	0.23	−0.65 ***	1.00					
PcA	0.48 *	0.05	0.13	0.39	−0.02	1.00				
pHbA	0.25	0.31	0.29	−0.07	0.44 *	−0.05	1.00			
SyrA	0.78 ***	0.12	0.24	0.13	0.15	0.64 ***	0.23	1.00		
SA	−0.11	0.23	0.32	−0.53 ***	0.49 *	−0.57 ***	0.21	−0.30	1.00	
Cat	0.17	−0.54 ***	−0.44 *	−0.22	0.17	0.01	0.26	0.51 *	−0.08	1.00

Abbreviations: TP—total phenols, FRAP—ferric reducing antioxidant capacity, CUPRAC—cupric reducing antioxidant capacity, DPPH—diphenyl picrylhydrazyl radical scavenging activity, Car—β-carotene, PcA—protocatechuic acid, pHbA—*p*-hydroxybenzoic acid, SyrA—syringic acid, SA—salicylic acid, Cat—catechin; * values of correlation coefficients significant at *p* < 0.05, *** values of correlation coefficients significant at *p* < 0.001.

**Table 4:** Content of selected macro- and micronutrients in the pulp of 18*Cucurbita* cultivars.

		Content, mg 100 g^−1^f.w.									µg 100 g^−1^f.w.	
Species	Cultivar	Ca	K	Mg	P	S	Na	B	Fe	Zn	Cu	Mn
***C. maxima***	Australian Butter	24.7 ± 0,1^ef^	218.8 ± 3.9 ^de^	14.2 ± 0.5 ^efgh^	49.9 ± 0.9 ^e^	17.8 ± 1.1 ^ef^	0.87 ± 0.05 ^bc^	0.20 ± 0.00 ^de^	0.25 ± 0.01 ^c^	0.37 ± 0.02 ^b^	59.1 ± 2.2 ^ef^	36.0 ± 1.7 ^d^
	Bambino	32.3 ± 0.2 ^g^	144.9 ± 6.9 ^a^	7.1 ± 0.9 ^ab^	19.3 ± 0.3 ^b^	15.6 ± 0.6 ^b^	0.41 ± 0.05 ^ab^	0.15 ± 0.02 ^bc^	0.24 ± 0.02 ^c^	0.30 ± 0.03 ^b^	53.8 ± 2.0 ^e^	33.9 ± 2.2 ^d^
	Buttercup	18.9 ± 0.7 ^bc^	303.6 ± 5.2 ^g^	13.3 ± 1.1 ^def^	54.0 ± 0.9 ^f^	26.4 ± 0.2 ^i^	2.22 ± 0.17 ^ij^	0.21 ± 0.00 ^de^	0.27 ± 0.04 ^cd^	0.56 ± 0.00 ^d^	86.2 ± 3.7 ^g^	34.0 ± 1.3 ^d^
	Chicago Warted Hubbard	24.5 ± 0.8 ^def^	324.8 ± 14.2 ^h^	7.3 ± 0.7 ^ab^	22.4 ± 0.8 ^c^	19.3 ± 0.1 ^g^	1.70 ± 0.06 ^gh^	0.18 ± 0.01 ^cd^	0.19 ± 0.00 ^b^	0.20 ± 0.01 ^a^	22.9 ± 1.9 ^a^	22.0 ± 1.4 ^c^
	Garbo	31.7± 1.7 ^g^	235.2 ±2.5 ^e^	10.8 ± 0.0 ^c^	23.2 ±1.7 ^c^	17.4 ± 0.4 ^def^	1.00 ± 0.09 ^cde^	0.24 ± 0.01 ^e^	0.17 ± 0.03 ^b^	0.19 ± 0.01 ^a^	25.4 ± 1.5 ^ab^	15.5 ± 1.1 ^ab^
	Hokkaido	26.1 ± 2.7 ^ef^	323.3 ± 0.4 ^h^	7.4 ± 0.5 ^b^	18.6 ± 0.3 ^b^	15.1 ± 0.4 ^c^	0.20 ± 0.02 ^a^	0.21 ± 0.02 ^de^	0.15 ± 0.02 ^b^	0.21 ± 0.00 ^a^	42.2 ± 0.6 ^cd^	14.5 ± 0.1 ^ab^
	Indomatrone	32.2 ± 1.2 ^g^	469.8 ± 10.8 ^k^	34.0 ± 0.3 ^i^	76.5 ± 1.4 ^h^	49.3 ± 0.2 ^k^	6.82 ± 0.33 ^m^	0.15 ± 0.02 ^bc^	0.47 ± 0.00 ^g^	0.88 ± 0.04 ^f^	122.8 ± 7.7 ^i^	103.5 ± 3.1^i^
	Triamble	22.5 ± 0.4 ^de^	194.9 ± 1.2 ^c^	13.6 ± 0.3 ^defg^	54.7 ± 0.1 ^f^	18.7 ± 0.1 ^fg^	2.62 ± 0.20 ^jk^	0.11 ± 0.00 ^ab^	0.28 ± 0.00 ^cd^	0.52 ± 0.02 ^cd^	68.9 ± 1.6 ^f^	38.6 ± 0.5 ^de^
***C. pepo***	Halloween	38.0 ± 2.3 ^h^	291.0 ± 3.4 ^g^	11.4 ± 0.1 ^cd^	23.8 ± 1.3 ^c^	15.4 ± 0.6 ^c^	1.51 ± 0.02 ^efg^	0.22 ± 0.01 ^de^	0.15 ± 0.00 ^b^	0.15 ± 0.01 ^a^	48.8 ± 0.7 ^de^	19.4 ± 1.6 ^bc^
	KamoKamo	20.8 ± 0.8 ^cd^	259.4 ± 1.9 ^f^	12.9 ± 0.6 ^cde^	24.0 ± 0.1 ^c^	17.2 ± 0.2 ^de^	2.40 ± 0.27 ^ij^	0.15 ± 0.00 ^bc^	0.31 ± 0.01 ^de^	0.35 ± 0.01 ^b^	52.0 ± 0.5 ^de^	34.8 ± 1.2 ^d^
	Miranda	17.8 ± 1.2 ^bc^	163.7 ± 8.8 ^b^	5.1 ± 0.8 ^a^	14.1 ± 1.1 ^a^	9.0 ± 0.1 ^b^	1.41 ± 0.12 ^defg^	0.14 ± 0.01 ^abc^	0.14 ± 0.00 ^b^	0.17 ± 0.01 ^a^	34.9 ± 2.0 ^bc^	11.4 ± 0.7 ^a^
	Sweet Dumpling	16.6 ± 1.7 ^b^	272.9 ± 3.5 ^f^	34.6 ± 1.0 ^i^	110.6 ± 0.8 ^j^	29.3 ± 0.8 ^j^	3.08 ± 0.03 ^k^	0.23 ± 0.00 ^e^	0.41 ± 0.02 ^f^	1.23 ± 0.04 ^g^	101.2 ± 3.3 ^h^	68.0 ± 3.4 ^g^
	Table Gold	24.8 ± 0.6 ^ef^	390.7 ± 0.7 ^i^	15.9 ± 1.8 ^h^	88.9± 0.5 ^i^	22.2 ± 0.8 ^h^	3.56 ± 0.17 ^l^	0.35 ± 0.02 ^f^	0.42 ± 0.01 ^f^	0.78 ± 0.05 ^e^	102.1 ± 6.1 ^h^	74.8 ± 5.7 ^h^
***C. moschata***	Butternut	34.4 ± 0.2 ^g^	212.3 ± 8.4 ^cd^	12.3 ± 0.4 ^cde^	30.9 ± 1.4 ^d^	15.7 ± 0.1 ^c^	1.09 ± 0.04 ^cdef^	0.15 ± 0.01 ^bc^	0.16 ± 0.01 ^b^	0.38 ± 0.03 ^b^	59.8 ± 1.7 ^ef^	32.5 ± 0.9 ^d^
	Kogigu	9.9 ± 0.5 ^a^	434.7 ± 6.8 ^j^	10.8 ± 0.1 ^c^	110.7 ± 2.5 ^j^	29.2 ± 0.3 ^j^	1.49 ± 0.30 ^efg^	0.14 ± 0.01 ^bc^	0.26 ± 0.00 ^cd^	0.46 ± 0.04 ^c^	148.4 ± 7.2 ^j^	43.3 ± 0.7 ^e^
	Musquéede Provence	27.0 ± 0.4 ^f^	222.9 ± 0.6 ^de^	15.3 ± 0.4 ^fgh^	65.3 ± 0.4 ^g^	16.0 ± 0.3 ^cd^	1.53 ± 0.27 ^fg^	0.09 ± 0.00 ^a^	0.27 ± 0.01 ^cd^	0.35 ± 0.00 ^b^	53.9 ± 4.2 ^e^	38.0 ± 0.8 ^de^
	Shishigatani	23.6 ± 0.3 ^def^	327.0 ± 4.9 ^h^	15.7 ± 0.4 ^gh^	87.9 ± 0.3 ^i^	21.9 ± 0.3 ^h^	2.09 ± 0.09 ^hi^	0.21 ± 0.03 ^de^	0.33 ± 0.01 ^e^	0.55 ± 0.03 ^d^	60.5 ± 3.7 ^ef^	60.9 ± 1.5 ^f^
***C. ficifolia***	Angel Hair	23.5 ± 0.7 ^def^	137.5 ± 4.8 ^a^	5.9 ± 0.2 ^ab^	12.9 ± 0.1 ^a^	7.5 ± 0.2 ^a^	0.97 ± 0.06 ^cd^	0.11 ± 0.01 ^ab^	0.08 ± 0.00 ^a^	0.13 ± 0.01 ^a^	15.4 ± 0.8 ^a^	34.6 ± 0.6 ^d^

The values are given as means ± standard errors, followed by the letters a–m (in superscripts) to indicate statistical significance. The values marked with the same letters in one column are not statistically different at the *p* < 0.05; *n* = 3; n.a., not analyzed.

**Table 5:** Eigenvalues, percentage of variance and cumulated percentage of variance for the first five principal components obtained in the PCA.

	Eigenvalue	% Variance	Cumulated % Variance
PC1	8.21	39.12	39.12
PC2	2.99	14.26	53.38
PC3	2.81	13.36	66.74
PC4	2.34	11.12	77.86
PC5	1.37	6.53	84.39

**Table 6:** Factor loadings of analyzed variables for the first five principal components.

Variable	PC1	PC2	PC3	PC4	PC5
Protocatechuic acid	−0.227	0.299	0.442	0.466	−0.466
*p*-hydroxybenzoic acid	−0.374	−0.470	0.292	0.384	0.311
Salicylic acid	−0.325	**−0.810**	−0.185	0.082	−0.137
Syringic acid	**−0.557**	0.491	0.196	0.536	0.069
B	−0.273	−0.177	−0.226	−0.123	**−0.837**
Ca	0.271	−0.689	−0.283	−0.139	−0.057
Cu	**−0.879**	0.157	0.025	0.242	−0.062
Fe	**−0.903**	−0.036	−0.232	−0.161	0.017
K	**−0.786**	−0.193	0.238	0.179	−0.325
Mg	**−0.817**	0.014	−0.296	−0.335	0.135
Mn	**−0.898**	−0.130	−0.138	−0.222	0.11
Na	**−0.810**	−0.261	−0.248	−0.174	0.178
P	**−0.854**	0.326	−0.110	0.133	−0.098
S	**−0.922**	−0.212	0.024	0.019	0.131
Zn	**−0.859**	0.157	−0.262	−0.269	0.008
Total phenols	**−0.740**	0.374	0.168	0.262	0.177
FRAP	−0.145	−0.048	**0.716**	−0.651	−0.052
CUPRAC	−0.452	−0.016	0.573	−0.571	−0.037
DPPH	0.102	0.573	0.381	−0.599	0.023
Soluble sugars	−0.077	−0.501	0.631	0.253	0.272
Amino acids	−0.127	−0.486	**0.741**	0.037	−0.149

Factor loading ≥ |0.70| are marked in bold.

## Supplementary Materials

The following are available online. Contain exemplary HPLC chromatograms of phenolic compounds identified in fruit extracts of four *Cucurbita* species; (Figure S1) and a table (Table S1) which depicts the coverage of the requirements for selected macro- and micronutrients by the consumption of 100 g servings of fresh pumpkin based on the RDA and AI values set for adults > 70 years old [71,72].
