# Supplementary material for: Antioxidants and Health-Beneficial Nutrients in Fruits of Eighteen Cucurbita Cultivars: Analysis of Diversity and Dietary Implications
Source: Molecules. 2020 Apr 14;25(8):1792. doi: 10.3390/molecules25081792 (PMC7221643; doi:10.3390/molecules25081792)
Supplement: Supplementary file 1 [file molecules-25-01792-s001.pdf]

Table S1. The coverage of the requirements for selected macro- and micronutrients by the consumption of 100 g servings of fresh *Cucurbita* fruits based on the RDA and AI values set for adults > 70 years old.

		Ca – % AI[*1]	Mg – % RDA[1]		Fe – % RDA[2]	Zn – % RDA[2]		Cu –% RDA[2]	Mn – % AI[2]	
**Species**	**Cultivar**		**women**	**men**		**women**	**men**		**women**	**men**
C. maxima	**Australian Butter**	2.1%	5.3%	4.0%	3.2%	4.6%	3.3%	6.6%	2.0%	1.6%
	**Bambino**	2.7%	2.7%	2.0%	3.0%	3.7%	2.7%	6.0%	1.9%	1.5%
	**Buttercup**	1.6%	5.0%	3.8%	3.3%	7.0%	5.1%	9.6%	1.9%	1.5%
	**Chicago Warted Hubbard**	2.0%	2.7%	2.1%	2.4%	2.6%	1.9%	2.5%	1.2%	1.0%
	**Garbo**	2.6%	4.1%	3.1%	2.1%	2.3%	1.7%	2.8%	0.9%	0.7%
	**Hokkaido**	2.2%	2.8%	2.1%	1.9%	2.6%	1.9%	4.7%	0.8%	0.6%
	**Indomatrone**	2.7%	12.8%	9.7%	5.9%	11.0%	8.0%	13.6%	5.8%	4.5%
	**Triamble**	1.9%	5.1%	3.9%	3.5%	6.5%	4.7%	7.7%	2.1%	1.7%
C. pepo	**Halloween**	3.2%	4.3%	3.3%	1.9%	1.9%	1.4%	5.4%	1.1%	0.8%
	**Kamo Kamo**	1.7%	4.9%	3.7%	3.9%	4.4%	3.2%	5.8%	1.9%	1.5%
	**Miranda**	1.5%	1.9%	1.5%	1.8%	2.1%	1.5%	3.9%	0.6%	0.5%
	**Sweet Dumpling**	1.4%	13.0%	9.9%	5.1%	15.3%	11.1%	11.2%	3.8%	3.0%
	**Table Gold**	2.1%	6.0%	4.5%	5.3%	9.7%	7.0%	11.3%	4.2%	3.3%
C. moschata	**Butternut**	2.9%	4.6%	3.5%	2.0%	4.7%	3.4%	6.6%	1.8%	1.4%
	**Kogigu**	0.8%	4.1%	3.1%	3.2%	5.8%	4.2%	16.5%	2.4%	1.9%
	**Musquée de Provence**	2.3%	5.8%	4.4%	3.4%	4.3%	3.1%	6.0%	2.1%	1.7%
	**Shishigatani**	2.0%	5.9%	4.5%	4.1%	6.9%	5.0%	6.7%	3.4%	2.6%
C. ficifolia	**Angel Hair**	2.0%	2.2%	1.7%	1.1%	1.6%	1.2%	1.7%	1.9%	1.5%

* RDA – Recommended Daily Allowance, the average daily dietary intake level that is sufficient to meet the nutrient requirements of nearly all (97 to 98 percent) individuals in a life stage and gender group.; AI – Adequate Intake, based on observed or experimentally determined estimates of average nutrient intake by a group (or groups) of healthy people. Source of RDA and AI values: 1 – [71], 2 – [72].

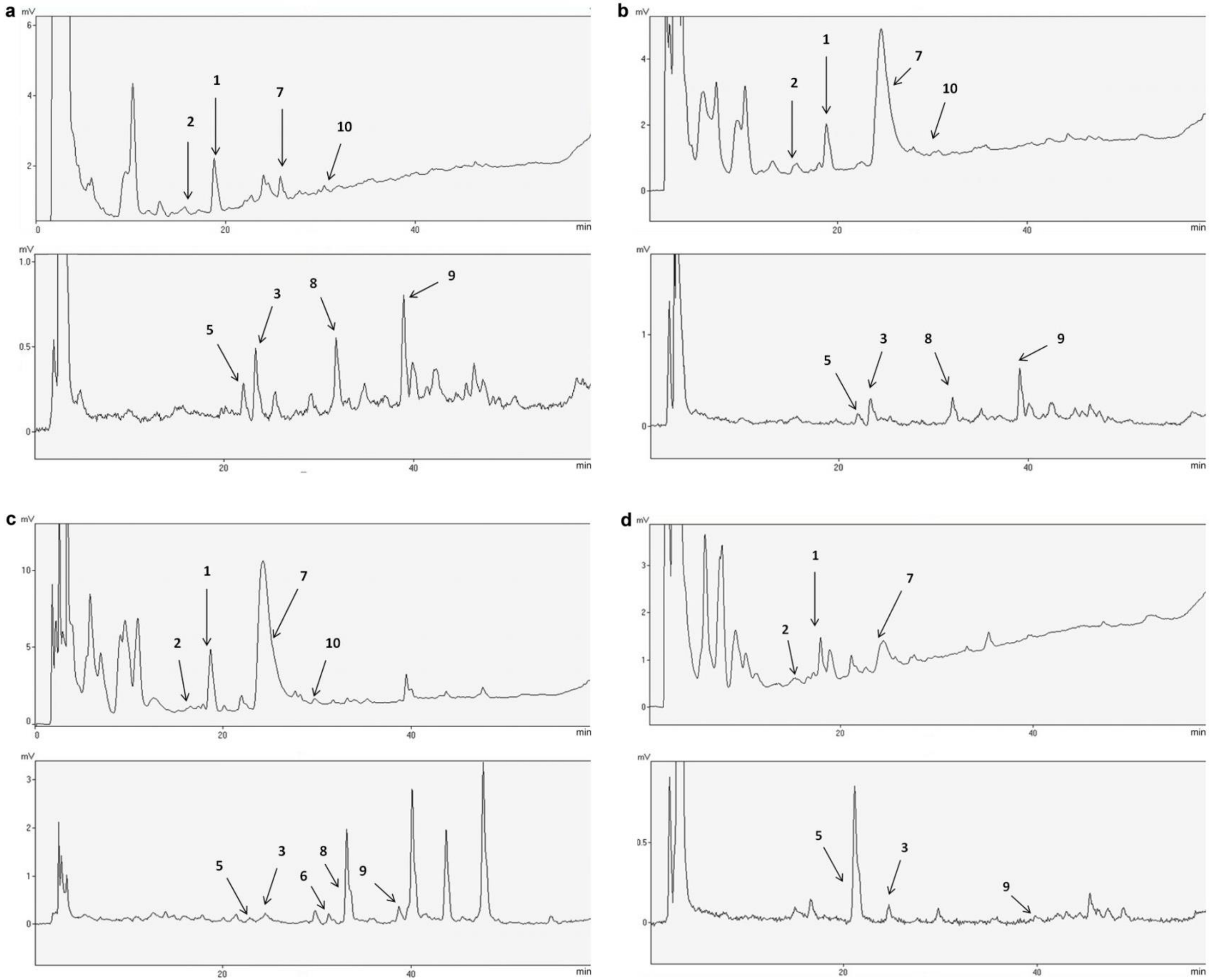

a
mV
min
1
2
7
10
5
3
8
9
b
mV
min
1
2
7
10
5
3
8
9
c
mV
min
1
2
7
10
5
3
6
8
9
d
mV
min
1
2
7
5
3
9
